# Judging Others by Your Own Standards: Attractiveness of Primate Faces as Seen by Human Respondents

**DOI:** 10.3389/fpsyg.2018.02439

**Published:** 2018-12-11

**Authors:** Silvie Rádlová, Eva Landová, Daniel Frynta

**Affiliations:** RP3 Applied Neurosciences and Brain Imaging, National Institute of Mental Health, Klecany, Czechia

**Keywords:** primates, facial attractiveness, visual perception, human preferences, uncanny valley, colors, visual cues

## Abstract

The aspects of facial attractiveness have been widely studied, especially within the context of evolutionary psychology, which proposes that aesthetic judgements of human faces are shaped by biologically based standards of beauty reflecting the mate quality. However, the faces of primates, who are very similar to us yet still considered non-human, remain neglected. In this paper, we aimed to study the facial attractiveness of non-human primates as judged by human respondents. We asked 286 Czech respondents to score photos of 107 primate species according to their perceived “beauty.” Then, we analyzed factors affecting the scores including morphology, colors, and human-likeness. We found that the three main primate groups were each scored using different cues. The proportions of inner facial features and distinctiveness are cues widely reported to affect human facial attractiveness. Interestingly, we found that these factors also affected the attractiveness scores of primate faces, but only within the Catarrhines, i.e., the primate group most similar to humans. Within this group, human-likeness positively affected the attractiveness scores, and facial extremities such as a prolonged nose or exaggerated cheeks were considered the least attractive. On the contrary, the least human-like prosimians were scored as the most attractive group. The results are discussed in the context of the “uncanny valley,” the widely discussed empirical rule.

## Introduction

Faces play a key role in the identification of other individuals, which is one of the most important skills needed in social communication of primates (Pascalis and Bachevalier, 1998; Santana et al., 2012). Humans can read emotional expressions from faces and gain a quick insight into the immediate mood of others (Ekman and Friesen, 1986; Fridlund, 1994; Russell, 1994; Calvo and Nummenmaa, 2016). Facial cues also bear information about the individual’s social role (age, sex, and race; for a review, see Yovel, 2016) or personality, such as dominance (Jones et al., 2010), extraversion (Borkenau et al., 2009), trustworthiness (Stirrat and Perrett, 2010), intelligence (Zebrowitz et al., 2002), or emotional stability (Penton-Voak et al., 2006).

Recognition of individual faces is so important that during evolution we gained a complex neural system specialized for just this function (Haxby et al., 2000; Zhao et al., 2018). Because of that, we are able to holistically distinguish faces that subtly differ in minimal position changes of inner facial features, i.e., the eyes, nose, and mouth (Maurer et al., 2002). We also use this recognition ability when evaluating the facial attractiveness: the evaluation is very strict as minimal deviation from the averageness or subtle distinctiveness can be perceived as unattractive or attractive. However, the more different the faces are from our own race and species, the more this ability weakens and diminishes. Using configural processing, humans can process same-race, conspecific faces with a higher success than faces of other races and species (Tanaka et al., 2004; Michel et al., 2006; Ge et al., 2009; Taubert, 2009), and the same applies for non-human primates: according to Gothard et al. (2009), macaques use configural processing when identifying faces of conspecifics, but turn to feature-based mode of analysis when processing pictures of human faces. The way in which humans consider attractiveness of faces of other species thus forms a very interesting question. In this matter, primates represent the perfect group to study—they include species phylogenetically closest to humans with very human-like faces, but also less similar species like the prosimians. Is it possible that human respondents see some primates as human caricatures and evaluate their facial attractiveness using the same facial cues as they use when evaluating facial beauty of conspecifics?

The majority of papers study facial beauty related to sexual attractiveness (for reviews, see Thornhill and Gangestad, 1999; Fink and Penton-Voak, 2002; Kościński, 2007). In this context, the best predictors for facial attractiveness are averageness (Jones and Hill, 1993; Rhodes and Tremewan, 1996; Komori et al., 2009; Zhang et al., 2011), symmetry (Grammer and Thornhill, 1994; Perrett et al., 1999; Scheib et al., 1999), sexual dimorphism (Perrett et al., 1998; Valenzano et al., 2006), smoothness of skin texture and color (Fink et al., 2001, 2006, 2012; Jones et al., 2004; Matts et al., 2007) and an absence of visible defects such as scars (Rankin and Borah, 2003) or congenital face clefts (Tobiasen, 1987).

In studies of human facial attractiveness, many aspects of the preferred facial traits vary under different domain specifity, i.e., different features may be preferred when considering facial attractiveness of short-term sexual partners and long-term romantic partners (DeBruine, 2005), competitors (Fisher, 2004), etc. Although the true nature of domain specifity that lies behind the ranking of primate facial “beauty” is unknown (possibly, the primates may be seen as rivals, cooperators, or may induce care-taking motivation), the respondents hardly evaluate the primates as potential romantic partners. However, recognition of human attractive and unattractive facial features is strongly tied to the identification of healthy and fertile mates and to the increase of one’s fitness (e.g., Thornhill and Gangestad, 1999; Fink and Penton-Voak, 2002; Little et al., 2011). One can thus imagine that the selection pressure led to the perfection of fast and precise ability to assess the attractiveness of the conspecific faces. The question of interest now is whether the cues for the recognition of attractive human faces remain the same when assessing the attractiveness of non-human, but similar faces. To answer this question, we examined various factors, often described as important cues in the evaluation of human faces, and analyzed their effect on human-perceived beauty of primate faces.

Instead of experimenting with subtle facial details using computer manipulations, we chose to examine the true facial variability of extant primate species, which is enormous. Some primates are more similar to humans than the others, and their facial features—the same features that are heeded in human facial attractiveness, i.e., eyes, nose, mouth, etc.,—are often exaggerated to the extremes that may be perceived as human caricatures. And while caricatures (i.e., faces with high distinctiveness, Deffenbacher et al., 1998) may be helpful for a better recognition of individuals (Rhodes et al., 1987; Mauro and Kubovy, 1992), it is the average human faces that are considered as attractive (e.g., Rhodes et al., 2001; Trujillo et al., 2014). Distinctiveness is only seen as attractive when composited from highly attractive features, such as the higher cheek bones, thinner jaws, larger eyes, shorter length between mouth and chin, and between nose and mouth (Perrett et al., 1994).

The sexual dimorphism, i.e., masculinity and femininity, also plays an important role in the perception of human facial attractiveness. For example, Little et al. (2002) found that women showed higher preference for male face masculinity when judging for short-term relationships than when judging for long-term relationships. When studying the effect of sexual dimorphism in non-sexual context, Little et al. (2008) found that both women and men preferred more feminine female faces and more masculine male faces, though the preferences were stronger in women than in men. Other papers (Perrett et al., 1998; Rhodes et al., 2000) report that both women and men preferred more feminine faces, regardless of the face gender. Most of the papers agreed on the lack of difference between male and female respondents in the direction of preferred sexual dimorphism, they only differed in the degree. However, masculine male and female faces are perceived by respondents of both genders as dominant (Perrett et al., 1998). The features that make faces look masculine or feminine are very specific. For example, larger jawbones, more prominent cheekbones, and thinner cheeks are all features of human male faces that differentiate them from female faces (Little and Hancock, 2002). However, the particular features may differ from species to species—e.g., masculine features of a Mandrill rather include elongated jaw and bright colors (Dixson, 2012). Thus, this variable is not fully comparable when studying facial attractiveness across all primates.

Apart from human facial attractiveness, a lot is known about the human-rated attractiveness of animals (e.g., Frynta et al., 2009; Marešová et al., 2009; Landová et al., 2012, 2018; Frynta et al., 2013, 2014; Lišková and Frynta, 2013; Lišková et al., 2015). Specific features, such as an overall shape, body size, achromatic components including pattern, surface (skin/feather/fur) texture and coloration, taxonomic classification and human-likeness, etc., determine whether an animal will be preferred or neglected. As the full variability of primate faces include those that are more and less similar to humans, a mix of factors usually known for affecting the attractiveness of both human faces and animals may play a role. Thus, many of these factors were included into the analysis.

In short, the purpose of this study is to examine human-perceived attractiveness (i.e., positive affinity toward an object) of primate faces. To our knowledge, this is the first study that focuses on the full variability of primate faces across all taxonomic groups. Other studies so far focused on facial attractiveness of humans or animals that are not closely related to humans (e.g., dogs and cats, Archer and Monton, 2011; Hecht and Horowitz, 2015; foxes, Elia, 2013). With the wide focus of this study, we aim to get insight into the human perception of primates, including the subjective recognition of human-animal boundary.

In our mainly exploratory study, we focused on the following two questions: (1) which factors determine the primate facial attractiveness (or beauty) rated by human respondents? (2) Do these determining factors differ among different primate groups? There are three main groups of primates: the prosimians, which are phylogenetically least related to us, the New World monkeys (Platyrrhini), and its sister taxon Catarrhini, which includes Old World monkeys, gibbons, great apes, and humans. Is the beauty of each of the groups rated using different cues? In search for the answers on these questions, we analyzed the effect of morphology, sexual size dimorphism (SSD), pattern, human-likeness, and colors, and we discussed the findings in terms of known facts about both human facial attractiveness and beauty of animals.

## Materials and Methods

### Selection of Species

There are about 376 known extant primate species (Wilson and Reeder, 2005) covering a wide range of morphological variability. For the purpose of this study, we aimed to choose a number of stimuli that would cover as much variability as possible. Thus, we included at least one species from each genus, except for *Phaner* (Fork-marked Lemur), *Procolobus* (Olive Colobus), *Pseudopotto* (False potto) and *Simias* (Pig-tailed Langur), of which there were no acceptable photographs available at the time of stimuli preparation. We also purposely excluded a human as we did not want to direct the respondents to rank the primate faces in the context of human facial attractiveness. The particular species within genera including similar species were selected based on availability of acceptable photographs. Where there was a high morphological variability within the genus, we included more species (two to eight; e.g., *Cercopithecus, Eulemur, Macaca, Saguinus*, etc.). The East Javan Langur (*Trachypithecus auratus*) was included in both black and orange forms. In case of sexually dimorphic species, only males were included. There is a trade-off between the inclusion of both sexes and taxonomic coverage as the number of stimuli need to be limited so that the respondents stay interested and give reliable rankings. Ten species were represented by two different individuals for a control. The random factors were set in a nested hierarchy. The variance of beauty ranking among individuals of the same species was negligible when compared to that between species (VarCorr function in R: Variance nested in the Group (infraorder/superfamily/family): 0.06898805; Genus: 0.10402177; Species: 0.24383308; Residual = individual: 0.05087559). We then assessed correlations between the conspecifics. Spearman’s correlations for all factors (colors, facial measurements, rankings) were high and significant at the *p* < 0.05 level, except for mouth width and chin (beauty: *r* = 0.73; human-likeness: *r* = 0.95). The dataset contained 117 pictures in total (107 after the removal of the control species/individuals, which were not included in the analyses to avoid pseudo-replication). For the full list of included species, see Supplementary Appendix 1.

### Preparation of the Stimuli

We collected good quality photographs of primates facing the camera. The main resources were Flickr^1^ or Wikimedia Commons^2^ licensed under the Creative Commons license. Supplementary resources were our own photos, photos provided by addressed authors, and books (Rowe, 1996; Mittermeier et al., 2010). For the full list of picture resources, see Supplementary Appendix 1.

**FIGURE 1:**
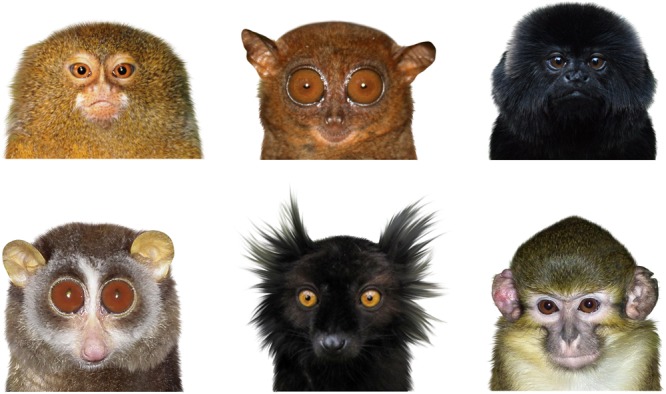
Examples of the stimuli rated by the respondents. The depicted primates are, from upper left to upper right: Pygmy Marmoset (*Cebuella pygmaea*), Philippine Tarsier (*Tarsius syrichta*), and Goeldi’s Monkey (*Callimico goeldii*); from lower left to lower right: Red Slender Loris (*Loris tardigradus*), Black Lemur (*Eulemur macaco*), and Northern Talapoin Monkey (*Miopithecus ogouensis*).

**FIGURE 2:**
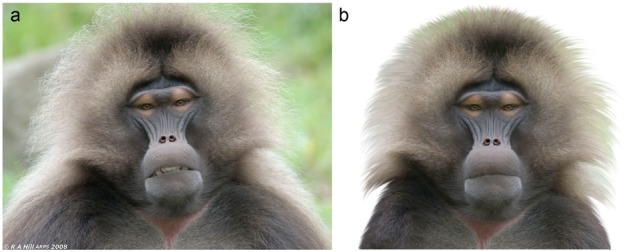
Example of a standardization/modification of the stimuli. **(a)** The original, unaltered picture of a Gelada (*Theropithecus gelada*). **(b)** We modified the photo to use it as a stimulus: the background was cut out, the head was rotated to a straight vertical position and the mouth was closed. Photo© Alan Hill, used with a permission.

Each photograph was modified to show the primate face in a standardized position: the background was cut off and set to white and the face (in the form of a bust, see Figure 1) was rotated so the eyes were intersecting a straight, notional horizontal line. The primate faces were size-adjusted to cover approximately the same space relative to each other on each image. When there were primates showing an emotional expression (e.g., a smile or a frown) or looking sideways, the photos were retouched so the face showed a neutral expression with eyes looking straight to the camera (see Figure 2). Because the used photos could not be standardized under the exact same angle, the primates in the pictures slightly differed in the degree of rotation on both vertical and horizontal axes. Thus, we could not test the effect of symmetry on human rankings of primate facial “beauty” as it clearly corresponded to the rotation of the faces. This rotation had no effect on any of the explained variables (none of the Spearman’s correlations were significant at the *p* < 0.05 level) and thus was excluded from further analyses.

### Definition of the Groups

**FIGURE 3:**
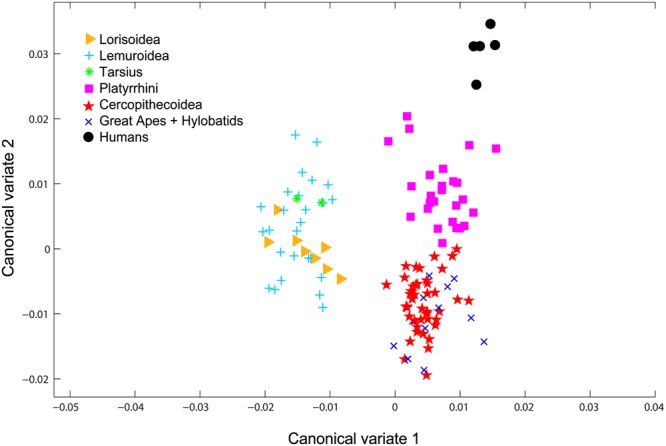
CVA analysis of the seven phylogenetic groups of primates. The analysis is based on the geometrical morphometry data measured from the primate photos and shows grouping into three main primate groups: the prosimians, Platyrrhini, and Catarrhini. Humans also form a separate group, but were excluded from the analyses.

The recognized taxonomy of Primates consists of seven distinct groups: Lorisoidea (African and Asian prosimians), Lemuroidea (Madagascar prosimians), the Tarsiers, Platyrrhini (New World primates), Cercopithecoidea (Old World monkeys), hylobatids (gibbons), great apes, and humans (Purvis, 1995; Yoder and Irwin, 1999; Pastorini et al., 2001; Geissmann et al., 2004; Mayor et al., 2004; Lei, 2008; Finstermeier et al., 2013). To identify groups of reasonable morphological variability suitable for the purpose of the analysis of human-rated facial attractiveness of primates, we performed the canonical variate analysis (CVA) using the geometrical morphometry data (see below, Section “Shape”). The CVA separated the primates into three distinct groups (see Figure 3), referred to as prosimians, Platyrrhini and Catarrhini in the manuscript. The analysis also confirmed morphological distinctiveness of humans when compared to other primates.

### Testing Human Preferences

Preferences for each of the primate faces were assessed using an online survey following Frynta et al. (2010) and Lišková and Frynta (2013). The respondents (*n* = 286, 91 men and 199 women) were Czech citizens, 15–69 years old (mean age was 22.7 years). Their task was to rate each of the faces on a scale (1–7 Likert scale; 1 = the most “beautiful,” 7 = the least “beautiful” or “ugly”) according to their perceived “beauty.” The photographs, resized to 360 × 540 pixels, were presented one by one on a computer screen in a random order. Prior to the presentation of the stimuli, the respondents were able to see the whole variability of the stimuli in the form of thumbnail-sized preview pictures (160 × 240 pixels). After that, the respondents started to score the pictures. The whole set was divided into groups of 39 photos, and after evaluating each of the groups, the respondents were allowed to take a rest, although the majority of the respondents finished the scoring without the need of a break. In total, all 286 respondents rated all 117 pictures.

### Explanatory Variables

#### Shape

**FIGURE 4:**
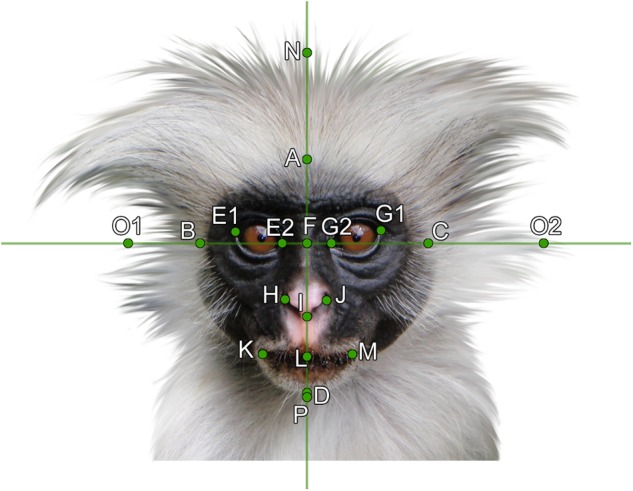
Facial landmarks used for the measurement of geometrical morphometry on the example of a Zanzibar Red Colobus (*Piliocolobus kirkii*). For the landmarks description, see text. Photo by Olivier Lejade via Wikimedia Commons, modified (see section “Materials and Methods”).

In our study, we aimed to cover the whole facial variability including the length of facial hair and the forehead size of animal (primate) faces. However, landmarks usually used in human facial studies either only include the shape and position of the eyes, nose, mouth, and chin (e.g., Mitteroecker et al., 2013), or include landmarks that are not applicable for frontal view of primate faces (e.g., Sforza et al., 2007). Thus, we adopted the landmarks of Borgi et al. (2014), who already defined landmarks of animal faces, which, with a few modifications, could easily fit our experimental stimuli (see Figure 4): (A) top of the head, (B) right side of the face, (C) left side of the face, (D) end of chin, (E1, G1) outer sides of right and left eyes, respectively, (E2, G2) inner sides of right and left eyes, respectively, (F) middle point of the reference cross, (H) right side of the nose, (I) left side of the nose, (J) tip of the nose, (K) left end of the mouth, (L) middle point of the mouth crossing the reference line, (M) right end of the mouth, (N) top point of head hair, (O1, O2) right and left tips of side hair, (P) tip of the chin hair (beard). Five human facial measurements were added (photos were selected randomly from the FEI Face Database; Thomaz, 2012) and these data were then used to perform the CVA analysis (see above in Section “Definition of the Groups”).

The landmarks were then converted to traditional morphometric variables: the face height (the A–D distance measured in pixels) and width (B–C), forehead height (A–F), eyes size (averaged E1–E2 and G1–G2 distances), nose length (F–I) and width (H–J), mouth width (K–M), side-hair (averaged O1–B and C–O2), top-hair (N–A), beard (D–P), interocular length (E2–G2), eyes-to-mouth distance (F–L), philtrum (nose-to-mouth distance, I–L), and chin (L–D). All analyzes and data transformations involving the landmarks were done using the IMP software series (Zelditch et al., 2012).

**FIGURE 5:**
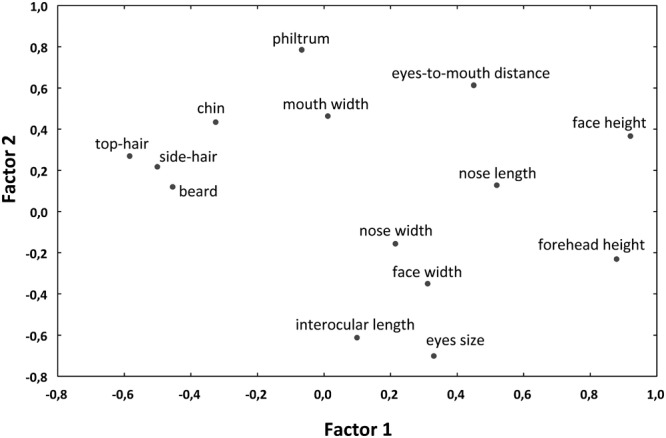
Plot of loadings of varimax normalized maximum likelihood factors, computed from morphometric traits of the primate pictures. The first extracted factor accounts for 20.5% of variation and can be interpreted as “outer facial features.” The second factor accounts for 22.5% of variation and corresponds to the “inner facial features.”

We then extracted maximum likelihood factors from these traits (varimax normalized) to reduce the number of morphological factors for the GLM/GLS analyses and especially to eliminate mutually correlated variables. The first extracted factor, accounting for 20.5% of variation, was interpreted as “outer facial features” (the height of the face and forehead on one side and the length of the beard and top-hair and width of the side-hair on the other side of the axis), while the second one (22.5%) corresponded to “inner facial features” (mainly the distance between eyes and nose from the mouth on one side and the size of the eyes and their distance on the other side of the axis; for factor loadings, see Figure 5).

#### Colors

To examine the effect of colors on the respondents’ ranking, we used the software Barvocuc (Rádlová et al., 2016) to extract specific information about hues, lightness and saturation of each of the stimulus pictures converted to the HSL colorspace. For a detailed description of the Barvocuc software, see (Lišková and Frynta, 2013 and Lišková et al., 2015). The variation in color is much smaller among primates than other animals, such as birds. Thus, the picture set included only the following colors, which we pre-defined using the software to describe the primate faces as accurately as possible: red (corresponding to the reddish brown in the primate photos) <350°; 18°), orange < 18°; 45°), yellow (corresponding to yellowish brown) <45°; 75°), and bluish tint <170°; 270°). The variability of blue color was too low in the dataset (only two primates possessed true blue facial parts: the Mandrill *Mandrillus sphinx* and Golden Snub-nosed Monkey *Rhinopithecus roxellana*). However, the blue color was present in a small amount on several photographs in the form of a bluish tint. Because blue color plays a crucial role in the determination of human preferences toward many groups of animals (Frynta et al., 2010; Lišková and Frynta, 2013; Lišková et al., 2015; Ptáčková et al., 2017), we decided to include the “bluish tint” color (blue hue minus the facial parts of *M. sphinx* and *R. roxellana*) as an explanatory variable for the analysis.

The values for saturation (S) and lightness (L) covered the interval 0–1. We defined three additional colors: black (*L* < 0.20), white (*L* > 0.71), and gray (*S* < 0.15). The white background of the stimuli was set to transparent and thus excluded from the calculation. In order to improve normality, the portion of colored pixels in the tested pictures was square-root arcsin transformed prior to the analyses. We also included the “pattern,” computed using an edge detection method (Sobel, 1978) as an explanatory variable in the analyses. The highest values of the pattern variable corresponded to the agouti coloration of some of the primates.

#### Sexual Size Dimorphism

Sexual dimorphism as studied in context of human facial attractiveness usually refers to the sexual shape dimorphism. This variable, however, is hardly comparable to primate facial sexual shape dimorphism. It is because each face represents a different species, and the particular features shaping males and females may differ for each species. These features may include various characteristics such as conspicuous cheeks, enlarged noses, colorful prolonged snouts, etc. These masculine features are not directly comparable to those of human males, which are defined by, e.g., subtle changes in jawbones size or more prominent cheekbones, as mentioned above (Little and Hancock, 2002). However, it is possible to use related species characteristics that is available from published sources—the sexual dimorphism in body size. Sexual selection, alongside with the increase in male body size, promotes the emergence of novel conspicuous traits, including those visible on primate faces. Thus, the larger the size difference between the sexes, the larger are the distinctive facial features. For example, size dimorphism in canines increases with SSD in primates (Leutenegger and Kelly, 1977; Kay et al., 1988) and thus modifies the primate mouth shape as bigger canines require more elongated jaws (Weston et al., 2004). Adult males of sexually dimorphic (male-larger) species display red and blue sexual skin (e.g., the Mandrill), capes of hair, and facial adornments (e.g., the Bald Uacari, Proboscis Monkey, Golden Snub-nosed Monkey, or the Orangutans; Dixson, 2012). In this paper, we utilized this variable to indirectly examine the effect of conspicuous traits on the human evaluation of primate facial attractiveness.

Sexual size dimorphism was expressed using the Lovich and Gibbons ratio (LG ratio; Lovich and Gibbons, 1992), which produces measures of sexual dimorphism continuous around 0. The values were computed as follows: (body weight of the larger sex/body weight of the smaller sex) -1, negative by convention when males are the larger sex and positive when females are larger than males. LG ratios of the primates set varied within the range of -1.371 in the Western Gorilla (*Gorilla gorilla*) to 0.313 in Lemurine Night Monkey (*Aotus lemurinus*). The body weights were adopted from Lindenfors and Tullberg (1998); Gordon (2006), and Mitteroecker et al. (2013).

#### Human-Likeness

Sixty respondents (different from the ones evaluating the attractiveness) repeated the procedure described above (Section “Testing Human Preferences”) to rate the primates’ human-likeness (1–7 Likert scale; 1 = most human-like, 7 = not human-like at all). Agreement among the respondents in human-likeness of the primates was exceptionally high. The intra-class correlation (ICC, see later in the text), assessed using a two-way, consistency measure, was in an excellent range: ICC = 0.986 for average-measure, 0.553 for single-measure (Hallgren, 2012). To ensure that the knowledge of the great apes being the most phylogenetically related to humans did not distort the overall agreement, we also checked for the ICC of the data excluding the Homoidea (great apes and gibbons): ICC was 0.983 for average-measure, 0.5 for single-measure; i.e., these analyses show that the respondents agreed well on the human-likeness of the particular primate groups/species and their rankings were not influenced by just the most human-like apes. The multivariate analysis of variance revealed no effect of gender, age, nor their interaction. Thus, we pooled the dataset and used the mean values in the analyses as a reliable estimate of human-likeness of the ranked primate species.

### Statistical Analyses

In order to quantify and test congruence in species ranking provided by different respondents, we adopted a two-way, consistency, average-measures intra-class correlation (ICC; McGraw and Wong, 1996; Hallgren, 2012) computed in R (irr package). Principal component analysis (PCA) was performed to visualize the multivariate structure of the data sets and to extract uncorrelated axes for further analyses. MANOVA and General Linear Models (LMs) were applied to test the effects of independent explanatory variables. Full LMs were further reduced according to Akaike criterion until log-likelihood tests revealed a significant comparison between the full and reduced models. Mann–Whitney test was used as a non-parametric alternative for variables deviating from normality (raw sores). The contribution of the explanatory variables (constrains) to the attractiveness rating of the primate faces was examined in redundancy analysis (RDA) as implemented in the R package vegan (Oksanen et al., 2017). RDA is a multivariate direct gradient method. It extracts and summarizes the variation in a set of response variables (subjective evaluation of primate beauty) that can be explained by a set of explanatory variables (see Section “Explanatory Variables”). This analysis permits to plot both response and explanatory variables to a space defined by the extracted gradients and enables to detect redundancy (i.e., shared variability) between sets of response and explanatory variables. Statistical significance of the gradients was confirmed by permutation tests. Most of the calculations were performed in R (R Development Core Team, 2010) and Statistica 9.1. (StatSoft, 2010).

### Ethics Statement

This study was carried out in accordance with the recommendations of Institutional Review Board (IRB), Faculty of Sciences, Charles University in Prague, approval no. 2013/7, with written informed consent from all subjects. All subjects gave written informed consent in accordance with the Declaration of Helsinki. The protocol was approved by the IRB.

## Results

### Agreement Among the Respondents

Results of the ranking procedure revealed considerable congruence among the respondents. Although the reliability of the individual rankings was quite low (ICC = 0.147, 0.204, 0.182 for men, women, and pooled data, respectively, with all *p* < 0.001), the ICC for the average-measures was in an excellent range: ICC = 0.940 for men, 0.981 for women, and 0.985 for the pooled data (Shrout and Fleiss, 1979; Cicchetti, 1994). These results indicate that there was a high degree of agreement within the group of the respondents and suggest that preferences for primate faces were rated similarly. Also the correlation between ranks provided by male and female respondents was very high: *r*^2^ = 81.6%, *p* < 0.001. Multivariate analysis of variance revealed no effect of age (Wilks = 0.6191, *F*_171, 109_ = 0.97, *p* = 0.575) or age × gender interaction (Wilks = 0.5823, *F*_171, 109_ = 1.13, *p* = 0.2436), nevertheless, a small, but significant effect of gender (Wilks = 0.5384, *F*_171, 109_ = 1.35, *p* = 0.041) was found. To identify the species that substantially contributed to the gender differences, we performed Mann–Whitney *U* tests comparing the raw ranks of each species in male and female respondents; the levels of significance were Bonferroni-corrected. Men significantly differed in their preferences from women in only five cases, all of which were preferred by men more than by women: de Brazza’s Monkey (*Cercopithecus neglectus*), Patas Monkey (*Erythrocebus patas*), Humboldt’s Woolly Monkey (*Lagothrix lagotricha*), Drill (*Mandrillus leucophaeus*), and the Sumatran orangutan (*Pongo abelii*). Because the gender differences were small and involved only 5 out of 107 examined species of primates, we decided to pool the genders in further analyses concerning the means or multivariate axes (RDA) computed from the preference ranks. Both of these methods extract the agreement among respondents and thus further blend the minor effect of gender.

### The Attractiveness

The primates whose faces were rated as the most “beautiful” were mostly prosimians: the top winners were the Black-and-white Ruffed Lemur (*Varecia variegata*), Ring-tailed Lemur (*Lemur catta*) and Southern Lesser Galago (*Galago moholi*). Moreover, little monkeys such as the marmosets (Callitrichinae) were favorite among the respondents, together with apes such as the Agile Gibbon (*Hylobates agilis*) and Bonobo (*Pan paniscus*). In contrast, the Proboscis Monkey (*Nasalis larvatus*) or the Bald Uacari (*Cacajao calvus*) were rated as the “least beautiful” (or “ugly”).

**FIGURE 6:**
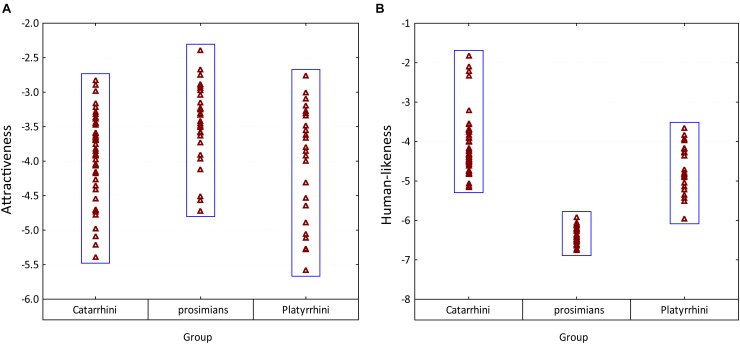
Variability in the mean rankings of **(A)** attractiveness and **(B)** human-likeness among three main groups of primates. Please note that the scales were inverted so that the higher value corresponds to higher attractiveness/human-likeness.

When overviewed within the particular groups, all three groups included both attractive and unattractive species (see Figure 6). However, the prosimians were rated as significantly more attractive than the other groups (*post-hoc* Tukey test, *p* < 0.01). The particular cues affecting the respondents’ decision and the relation to the uncanny valley theory is discussed below in the respective sections.

#### RDA Analysis of the Factors Affecting Attractiveness

We employed RDA to examine the contribution of various explanatory variables to the ratings of primate facial attractiveness. We utilized the automatic model-building feature based on both Akaike criterion (but with permutation tests) and on permutation *p*-values. Both methods agreed on the inclusion of the following variables into the reduced model, which were then confirmed as significant by the sequential “Type I” test (*n* permutations = 10,000): Factor2 (i.e., inner facial parts; *F*_1,100_ = 22.4244, *p* < 0.0001), human-likeness (*F*_1,100_ = 6.0119, *p* < 0.0001), blue color (*F*_1,100_ = 3.4310, *p* = 0.0011), Factor1 (i.e., outer facial parts; *F*_1,100_ = 2.8690, *p* = 0.0050), LG (*F*_1,100_ = 2.3811, *p* = 0.0125), and pattern (*F*_1,100_ = 1.9238, *p* = 0.0400). The RDA model has generated six constrained axes, which explained 28.08% of the full variability.

**FIGURE 7:**
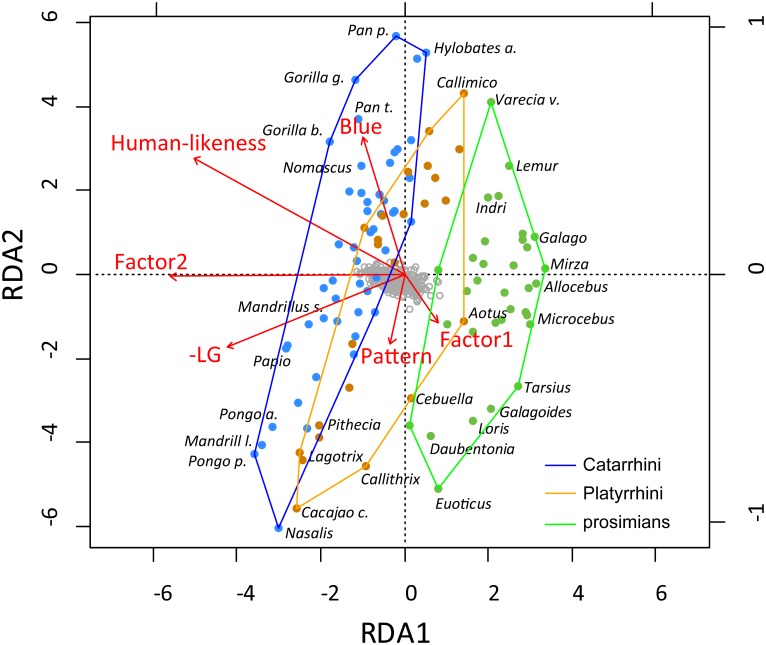
RDA analysis of the factors affecting primate facial attractiveness. RDA1 correlates with Factor2, i.e., the inner facial parts, and RDA2 axis corresponds to the actual attractiveness (see text). The graph shows that the respondents’ classification of the primate facial beauty differs among the groups and is mainly based on the inner facial properties of the species.

The visualization of the RDA results (see Figure 7; note that for a better clarity, we multiplied human-likeness and LG by -1 so the higher the number, the higher is both the human-likeness and exaggeration of the male facial parts) showed that Factor2, i.e., the inner facial parts, dominated the first multivariate axis (RDA1; correlation of RDA1 site scores with Factor2: *r*^2^ = 72.2%, *p* < 0.0001). As the most attractive species are located on top and the least attractive on the bottom of the graph (second axis), we can conclude that the RDA2 axis corresponds to the actual attractiveness of the species. Correlation of the mean attractiveness scores with the RDA2 site scores supports this: *r*^2^ = 69.6%, *p* < 0.0001. The only factors associated with this attractiveness irrespective of the second axis (and thus the primate grouping, which corresponds to this axis) are blue color (positive effect) and pattern (negative effect). The graph clearly shows that the grouping of the primates (based on real morphology) reflects the respondents’ ratings of the species’ beauty, i.e., the respondents’ classification of the primate facial beauty differs among the groups and is mainly based on the inner facial properties of the species. Both the extent of human-likeness and the extent of male sexual dimorphism (-LG) feed this second morphological axis.

#### GLM of the Factors Affecting Attractiveness

In order to examine which factors contribute to the variability of preference rankings, we performed LMs (see Table 1). The initial full model of all the primates together (*n* = 107) included the group, outer (Factor1) and inner (Factor2) facial features, LG, human-likeness, mean lightness, pattern, mean saturation, reddish brown, orange, yellowish brown, and bluish tint. After reduction using the Akaike Information Criterion (AIC, Akaike, 1998), the reduced model explained 34.4% of variation in preference ranks (*p* < 0.0001) and included the group *F*_2,100_ = 11.0290, *p* < 0.001), inner facial features (*F*_1,100_ = 16.2674, *p* = 0.0001), LG (*F*_1,100_ = 5.7186, *p* = 0.0187), human-likeness (*F*_1,100_ = 8.0299, *p* = 0.0056), and bluish tint (*F*_1,94_ = 9.4351, *p* = 0.0027).

**Table 1:** The final reduced models (GLM) describing the effect of the morphology, colors, and human-likeness on the attractiveness scoring of each of the main primate groups.

	ANOVA			Coefficients			
	*Df*	*F*	Pr (>*F*)	Estimate	SE	*t*	Pr (>|*t*|)
**(a) All primates (attractiveness, *r*^2^ = 0.3437)**
(Intercept)				2.7457	0.4545	6.0420	0.0000
Group	2	11.0290	0.0000				
Group (Platyrrhini)				0.2927	0.1742	1.6800	0.0961
Group (prosimians)				–0.2936	0.2743	–1.0710	0.2869
Factor2	1	16.2674	0.0001	0.3488	0.1238	2.8180	0.0058
LG	1	5.7186	0.0187	–0.5119	0.2198	–2.3290	0.0219
Human-likeness	1	8.0299	0.0056	0.2141	0.0987	2.1690	0.0325
Bluish tint	1	9.4351	0.0027	–1.5962	0.5196	–3.0720	0.0027
Residuals	100						
**(b) Catarrhini (attractiveness, *r*^2^ = 0.5461)**
(Intercept)				2.5497	0.3633	7.0170	0.0000
Factor2	1	27.1710	0.0000	0.4386	0.1109	3.9540	0.0003
LG	1	5.5832	0.0226	–0.2683	0.1959	–1.3700	0.1777
Human-likeness	1	16.6525	0.0002	0.2576	0.0819	3.1450	0.0030
Reddish brown	1	2.1168	0.1528	0.8435	0.5552	1.5190	0.1359
Bluish tint	1	12.4338	0.0010	–1.4408	0.4086	–3.5260	0.0010
Residuals	44						
**(c) Platyrrhini (attractiveness, *r*^2^ = 0.4006)**
(Intercept)				5.2577	1.5350	3.4250	0.0032
LG	1	3.4340	0.0813	–1.4253	0.7377	–1.9320	0.0702
Human-likeness	1	0.2547	0.6203	–0.7377	0.3366	–2.1920	0.0426
Mean lightness	1	6.8828	0.0178	3.7882	1.2575	3.0130	0.0078
Pattern	1	3.6745	0.0722	2.9365	1.5838	1.8540	0.0812
Orange	1	0.6811	0.4206	0.5885	0.4672	1.2590	0.2249
Yellowish brown	1	6.4430	0.0212	–1.7697	0.6972	–2.5380	0.0212
Residuals	17						

We then conducted the same analysis separately for each of the groups Catarrhini, Platyrrhini, and prosimians (see Table 1b,c). Catarrhini (*n* = 50): the reduced model (*r*^2^ = 54.6%, *p* < 0.0001) included the inner facial features (*F*_1,44_ = 27.1710, *p* < 0.0001), LG (*F*_1,44_ = 5.5832, *p* = 0.0226), human-likeness (*F*_1,44_ = 16.6525, *p* = 0.0002), reddish brown, and bluish tint (*F*_1,44_ = 12.4338, *p* = 0.0010). Platyrrhini (*n* = 24): the LG, human-likeness, mean lightness, pattern, orange, and yellowish brown color remained in the model (*r*^2^ = 40.1%, *p* < 0.0001), but only the mean lightness (*F*_1,17_ = 6.8828, *p* = 0.0178) and yellowish brown (*F*_1,17_ = 6.4430, *p* = 0.0212) retained significance. The model for prosimians (*n* = 33) failed to explain any variability and was not significant.

## Discussion

### The Effect of Shape

The inner facial parts represent one of the strongest factors determining the beauty of the species within the group of the primates most similar to us, i.e., the Catarrhini. Thus, the size of eyes, interocular length, mouth width, and length from the nose to the mouth (or eyes to the mouth) are strong cues that our respondents use as a guide when ranking the “beauty” of Catarrhine faces. Consequently, although the respondents were not instructed to categorize the primates (the scoring procedure in our experiment instructed the respondents to assign scores to the pictured faces according to the subjectively perceived beauty), the RDA2 axis shows that the respondents still categorized the ranked subjects, and this categorization was mainly based on the inner facial parts of the primate faces. This phenomenon is often reported in studies focused on human perception of animal beauty (e.g., snakes; Marešová et al., 2009; Landová et al., 2012; birds: Lišková et al., 2015) and resembles the task recognized as unsupervised human categorization (Pothos and Chater, 2002; Pothos and Close, 2008).

In literature, the understanding of the role of inner and outer facial features is unclear. Some authors claim that young children mostly use the outer facial features as the cues for facial recognition, and then this pattern switches to the “adult version,” in which the faces are recognized using the inner features (Campbell et al., 1995; Turati et al., 2006). Other authors argue that both children and adults use inner facial features for the recognition of familiar faces, but outer facial features for recognition of the unfamiliar ones (Ellis et al., 1979; Want et al., 2003; Bonner and Burton, 2004; Ge et al., 2008). Our results show that the inner facial features are not only used for categorization of the primates, but also play a very important role in the assessment of the facial beauty of the Catarrhine primates. Outer facial features are used to a much less extent, but also appear to contribute to the assessment of primate beauty (see Figure 7).

### Colors and Pattern in Primate Facial Attractiveness

Our results show that two colors affect the attractiveness of primate faces: the bluish tint (in Catarrhines and the full picture set) and the yellowish brown color (Platyrrhines). In literature, colors do play a role in the assessment of attractiveness, especially the red color, which is important for both humans and non-human primates. Human faces exhibiting brighter red are perceived as healthier and more attractive (Re et al., 2011). Female Rhesus Macaques prefer males with redder faces (Waitt et al., 2003; but see Waitt et al., 2006, where this preference only applied to red hindquarters). Moreover, red clothing or even extraneous red (for example, red background of a presented picture stimulus) is perceived as more attractive by both human respondents (Elliot et al., 2010) and non-human primates (Hughes et al., 2015).

We examined the full variability of colors present in the picture set of primates, i.e., not only red, but also orange, yellow, and the bluish tint. Within our examined picture set, only three primates possessed bright red coloration of the face (The Bald Uacari, Silvery Marmoset, and Japanese Macaque). Thus, we instead tested the effect of the overall presence of the red color, mostly expressed as darker red or reddish brown fur color. However, we found no effect of this color on human preferences. The only color that positively affected human decisions toward all primates (regardless of the particular groups) was blue—the same color that is, within the context of facial attractiveness, usually perceived negatively, as blue, pale faces indicate low oxygenation and poor health (Stadie, 1919). However, blue is very often reported as the most preferred color in other studies examining human rating of animal beauty, e.g., parrots (Frynta et al., 2010), birds (Lišková and Frynta, 2013; Lišková et al., 2015), and even snakes (Ptáčková et al., 2017). In the latter case, the color was present only in the form of a bluish tint. Similarly, in this paper, this bluish tint affected the overall evaluation of the beauty and was rather the effect of the photos than the primate species themselves.

When analyzing the particular groups, the GLM revealed that the attractiveness of the New World monkeys was positively affected by the yellowish brown color. This is in agreement with the perception of human faces as more attractive (Stephen et al., 2012) and healthier (and thus more attractive: Stephen et al., 2009) show that the respondents increased yellow color together with red and overall lightness when aiming to create a healthy-looking human face. Even papers dealing with animal attractiveness report preference for yellow color in some cases, especially when rating the beauty of birds (Lišková and Frynta, 2013; Lišková et al., 2015). However, animal attractiveness is usually mainly determined by the pattern and achromatic contrasts (Lišková and Frynta, 2013; Lišková et al., 2015). Similarly, the only other variable next to the yellow color that explained the attractiveness ratings of the New World primates was the mean lightness—the respondents rated darker monkeys as more beautiful. This agrees with the animal studies but contrasts human-facial studies in which either lighter (Van den Berghe and Frost, 1986; Stephen et al., 2009; Coetzee et al., 2012) or medium-toned, but not pale or black faces are rated as more attractive (Frisby, 2006; Stephen et al., 2012, but see Fink et al., 2001).

Although the pattern variable was dropped out from the final LMs, the multivariate analysis shows that, at least to some extent, it negatively affects the evaluation of overall primate facial attractiveness. This seems to contradict some of the papers that report positive effect of pattern to the evaluation of animal (Lišková et al., 2015; Ptáčková et al., 2017) and mammalian (Landová et al., in prep.) beauty. However, it is the highly contrasting pattern of large spots, stripes, and other marks, that positively affects the perceived animal beauty; not the diminutive unevenness of the fur color (i.e., agouti-type fur coloration), which is what the pattern variable used in this study corresponded to and which was perceived negatively. The primates possessing contrasting patches of fur coloration (e.g., the Black and White Ruffed Lemur and the Ring-tailed Lemur) were still considered the most beautiful.

Homogenous skin color distribution and surface topography (wrinkles), signs of health and age, also affect human preferences for attractive conspecific faces (Samson et al., 2010) and could possibly affect the preferences of primate faces as well. However, our set of stimuli was not controlled for the age of the depicted individuals (they were all adults of unspecified age) and thus we could not test the effect of the features affecting perception of age. Moreover, majority of the species included in the study possessed a face that was fully covered by fur. A carefully designed experiment with more uniform stimuli varying only in facial surface topography (e.g., faces of chimpanzees of varying age, or a manipulated picture set) would be needed to examine this interesting question.

### SSD, Averageness, and Facial Extremities

There is not much variability within the prosimians in SSD, as most species have genders of similar size (Dixson, 2012). Platyrrhine primates differ more (variance in LG reaches from -0.72 in the Brown Capuchin to 0.31 in the Lemurine Night Monkey), but still lack the most prominent facial extremities typical for many male-larger Catarrhine species such as the Western Gorilla, Mandrill, Drill, Orangutans, Golden Snub-nosed Monkey, Proboscis Monkey, Patas Monkey, Gelada, or the Lion-tailed Macaque. Thus, it is not surprising that the degree of sexual dimorphism only affects the beauty within the Catarrhines—the male-larger species are perceived as less beautiful. In other words, the respondents rate conspicuous facial features (“extremities”) negatively. Similarly, many researchers agree that distinctive features (caricatures) usually help for better recognition of individual faces (Rhodes et al., 1987; Mauro and Kubovy, 1992; Lee et al., 2000; but see Hancock and Little, 2011), but are rated negatively when being evaluated for attractiveness (Deffenbacher et al., 1998). In other words, conspicuous features are usually rated as unattractive as opposed to average features (e.g., Rhodes and Tremewan, 1996; O’Toole et al., 1999). However, preference for facial averageness may be based on a more general principle as other objects such as dogs, birds, fish, or cars were also found to be preferred when they were of an average shape (Halberstadt and Rhodes, 2000, 2003).

### Human-Likeness and the Uncanny Valley Theory

The uncanny valley theory describes an empirical rule first mentioned in an essay by a robotics professor Masahiro Mori in 1970 (and later re-published in English for a broader audience to see; Mori et al., 2012). Mori hypothesized that if a robot resembled humans in appearance, people would feel affinity toward it, up to the point where it was too similar to humans—almost undistinguishable. At that point, people would experience a negative, eerie-like sensation, and he called this descent of affinity the “uncanny valley.” This relationship of human-likeness and attractiveness (in sense of positive affinity toward an object; for a relationship between the emotional ratings of eeriness and attractiveness, see Burleigh et al., 2013) was later tested in a number of papers, which found evidence in support of the uncanny valley theory (e.g., Seyama and Nagayama, 2007; MacDorman et al., 2009; Mitchell et al., 2011). Steckenfinger and Ghazanfar (2009) described a support for this phenomenon even in macaque monkeys, which preferred realistic and stylized macaque faces over faces very close to realism. The reason behind uncanny valley is unclear; often disputed mechanisms include the atypical feature hypothesis or the category conflict hypothesis (Burleigh et al., 2013). In the first case, the effect of uncanny valley is present when evaluating pictures that include an abnormal feature, such as bizarre eyes (Seyama and Nagayama, 2007). In the second case, the uncanny valley negatively affects stimuli containing features belonging to multiple categories, eliciting discomfort because it is evaluated as ambiguous and confusing (Saygin et al., 2011). Once the respondents cease to recognize the conflicting object as “human,” the attractiveness returns back to the linear character of the observed attractiveness (or eeriness).

In this matter, our results may seem contradictive as overally the most preferred primates were the prosimians, which were rated as the least human-like. However, within the group of Catarrhine primates, i.e., the group phylogenetically closest to humans (which also includes species most similar to humans, see Figure 6B), human-likeness positively affected the rated facial attractiveness. Thus, it is reasonable to examine whether the effect of uncanny valley can be applied to the results within this group. The graph clearly shows that some of the most human-like species do “fall into the valley,” but when looked at in more detail, there are exceptions to this uncanny valley rule. Some primates sharing similar rates of human-likeness fall into the notional valley, while others remain attractive. The unattractive, yet human-like primates, are represented by species such as the Orangutans, Proboscis Monkey, and the Drill, and the reason may be the presence of the abnormal (distinctive) features, as discussed above in Section “SSD, Averageness, and Facial Extremities”. Interesting fact is that at least some of these features, such as the prolonged nose of the Proboscis monkey, are not perceived as unattractive *per se*: for example, the elephants, elephant shrews, coatis or tapirs all possess a prolonged nose, but they were all rated as very or fairly attractive in a previous study (Frynta et al., 2013). Thus, these results cannot be interpreted simply as a preference for average feature size, but rather as a preference for average feature size when present on a human or human-like animal. Possibly, our complex neural system for facial recognition causes the judgements of “beauty” to be far more strict when judging “humans” (including human-like objects and animals) than different objects (Hanson, 2005). The uncanny valley phenomenon can be in fact linked to the expertise to human faces: the reason why the uncanny effect is so widespread is because every human has an expertise in recognizing humans; however, it is possible that the same effect might be observed in other types of expertises, as these display similar behavioral and neuropsychological pattern (Diamond and Carey, 1986; Xu, 2005; Dufour and Petit, 2010).

**FIGURE 8:**
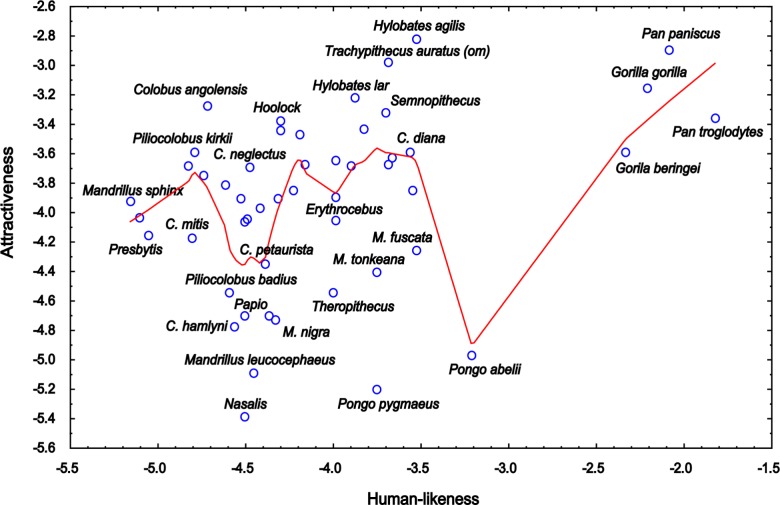
Relationship between the mean rankings of attractiveness of the Catarrhine primate faces and the mean rankings of their human-likeness (the scales were inverted so that the higher value corresponds to higher attractiveness/human-likeness). The LOWESS (Locally Weighted Scatterplot Smoothing) fitting line shows that an “uncanny valley” exists within human-like primates, but not within the ones almost fully human (Gorillas and Chimpanzees). However, many other “overcome the valley” because primates rated as fairly human-like can be rated as either attractive or unattractive depending on their traits (see the text).

Previously, some researchers showed that even distinct, extreme features (up to some point) can be perceived as more attractive than average, if the exaggeration is based on *attractive* features. Such feature then may represent a super-stimulus, which is a concept derived from ethological studies, describing an object that contains features more accentuated than natural stimulus, which elicits a response more strongly than the stimulus for which it evolved (Tinbergen, 1951). For example, Perrett et al. (1994) report that enhanced female features, including higher cheek bones, thinner jaws, and larger eyes, were rated as more attractive than average (also see Perrett et al., 1998), and similar results were found by Jones and Hill (1993), who found that enlarged proportion of eye width to face height (i.e., feminine/neotenous feature) was preferred more than the average proportions. Could the presence of enhanced attractive features be the reason why in our data, some human-like primates are attractive above the linear relationship between human-likeness and attractiveness (see Figure 8)? For example, the Agile Gibbon (*H. agilis*) does have a contrasting, black-and-white face, a feature found to be very attractive in human preferences for animals (Frynta et al., 2013; Lišková et al., 2015). It would be interesting to examine this phenomenon in another study with more controlled, manipulated stimuli.

The support for the uncanny valley theory is ambiguous in our study. Some of the most human-like primates do fall into the notional “valley”; however, some other primates overcome it. Thus, our results rather show that it is the “atypical feature present on a human-like object” that makes some of the primates to be rated very negatively. However, this does not necessarily neglect the uncanny valley. Rather, it supports the atypical feature hypothesis of the uncanny valley theory (Seyama and Nagayama, 2007; MacDorman et al., 2009).

## Conclusion

In our study, we focused on human evaluation of primate facial attractiveness. We found that there are differences in the evaluation of the three main primate groups. The attractiveness of the Catarrhine primates, i.e., the Old World monkeys, Gibbons, and the Great Apes, was explained by human-likeness, and also by factors similar as those usually utilized when evaluating human facial attractiveness: the inner facial features and SSD (i.e., lack of extreme, conspicuous features). Interestingly, the proportions of inner facial features were only used when evaluating the most human-like primates; in other groups, this factor had no effect, and its importance thus cannot be attributed to the evaluation of faces in general, but only those resembling humans.

The Platyrrhine primates, i.e., the New World monkeys, are phylogenetically more distant to humans. Regarding similarity to humans, they are somewhere between the Catarrhines and the prosimians (see Figure 6), and the results explaining their attractiveness scores reflect this. Their attractiveness is determined by human-likeness, yellowish brown color, and the mean lightness. However, the respondents liked more the monkeys that were scored as less-human like. The orange color, pattern, and SSD are all factors that could not be excluded from the final model but showed to be insignificant. The number of Platyrrhine primates included in the analysis was small though and it is thus possible that a larger sample could reveal significance of these factors. One way or another, the attractiveness of the Old World monkeys seem to be affected by factors that are otherwise reported as affecting evaluation of both human and animal attractiveness. On the contrary, the prosimians were rated as the most beautiful, but our analysis failed to reveal the particular cues responsible for their high scores.

## Data Availability Statement

The datasets generated during and/or analyzed during the current study are available in the Mendeley repository, http://dx.doi.org/10.17632/ssv9m953mb.1.

## Author Contributions

DF, SR, and EL conceived and designed the research and interpreted the results. SR performed the research and wrote the paper. DF and SR analyzed the data.

## Conflict of Interest Statement

The authors declare that the research was conducted in the absence of any commercial or financial relationships that could be construed as a potential conflict of interest.

## Footnotes

1 https://www.flickr.com/

2 https://commons.wikimedia.org
